# Anticancer therapy-induced adverse drug reactions in children and preventive and control measures

**DOI:** 10.3389/fphar.2024.1329220

**Published:** 2024-02-15

**Authors:** Hui Yan, Penggao Wang, Fang Yang, Weyland Cheng, Congcong Chen, Bo Zhai, Yang Zhou

**Affiliations:** ^1^ Henan Provincial Clinical Research Center for Pediatric Diseases, Henan Key Laboratory of Pediatric Genetics and Metabolic Diseases, Children’s Hospital Affiliated to Zhengzhou University, Henan Children’s Hospital, Zhengzhou Children’s Hospital, Zhengzhou, China; ^2^ Department of Cardiothoracic Surgery, Children’s Hospital Affiliated to Zhengzhou University, Henan Children’s Hospital, Zhengzhou Children’s Hospital, Zhengzhou, China

**Keywords:** adverse drug reactions, anticancer drugs, prevention, measures, children

## Abstract

In recent years, considerable achievements have been made in pediatric oncology with the innovation and development of antitumor drugs. However, compared to adults, children as a special group have not yet matured fully in terms of liver and kidney function. Moreover, pediatric patients are prone to more adverse drug reactions (ADRs) from the accumulation of antineoplastic drugs due to their smaller body size and larger body surface area. Chemotherapy-related ADRs have become a non-negligible factor that affects cancer remission. To date, studies on ADRs in pediatric cancer patients have emerged internationally, but few systematic summaries are available. Here, we reviewed the various systemic ADRs associated with antitumor drugs in children and adolescent patients, as well as the advances in strategies to cope with ADRs, which consisted of neurotoxicity, hematological toxicity, cardiotoxicity, ADRs of the respiratory system and gastrointestinal system and urinary system, ADRs of the skin and its adnexa, allergic reactions, and other ADRs. For clinicians and researchers, understanding the causes, symptoms, and coping strategies for ADRs caused by anticancer treatments will undoubtedly benefit more children.

## 1 Introduction

Malignant tumors are serious diseases that threaten the health and lives of children. It was reported that more than 1.3 million children and adolescents were annually diagnosed with cancer worldwide (YOU et al., 2021). The incidence rate of developing cancer ranged from 130 to 160 cases per million children, and the incidence had been increasing at a rate of about 2.8% per year for the past 10 years (GUPTA et al., 2020a). As a global public health crisis, childhood tumors have become the second leading cause of childhood death, second to accidental deaths (STELIAROVA-FOUCHER et al., 2017). From 2010 to 2020, the incidence rate of childhood tumors was 8.71/100,000 and the mortality rate was 3.63/100,000 in China, according to the data from the National Cancer Center of China (ZHENG et al., 2015). The first global burden of disease (GBD) assessment of childhood and adolescent cancer using disability-adjusted life-years (DALYs) showed that childhood cancer had become the sixth largest cancer burden in the world (The global burden of childhood, 2019). Thus, it is urgent to focus on the issue of childhood cancers.

With the development of antitumor drugs in recent years, significant achievements have been made in the treatment of childhood cancers. The 5-year survival rate of children under 14 years old with tumors was 84.0% according to U.S. statistics in 2021, with the 5-year survival rate of children with lymphoid leukemia, Hodgkin lymphoma and non-Hodgkin lymphoma (including Burkitt) exceeding 90.0% (SIEGEL et al., 2021). Unfortunately, unlike adults, pediatric patients are more susceptible to ADRs because of the special characteristics of children. Firstly, children have a smaller body size and a larger proportion of body surface area, which makes it easier for drug accumulation to occur in the body. Secondly, the growth and development process of children is a combination of many highly complex organ maturation processes, and these parallel developmental processes follow different temporal developmental trajectories, so that the absorption, distribution, metabolism, excretion and drug responsiveness of children to drugs are different from those of adults. Finally, children’s bodies are at a stage of growth and development where the blood-brain-barrier (BBB) is still imperfect, and metabolic organs such as the liver and kidneys are not yet mature, and the distribution and activity of drug-metabolising enzymes/transporters are poorer than those of adults, so their tolerance and responsiveness to drugs are different from adults. The ADRs caused in children are multifaceted, such as hematopoietic suppression, gastrointestinal reactions, and neurotoxicity (BOND et al., 2008; PAWAR et al., 2018). ADRs refer to the harmful and unrelated to the purpose of medication that occur when a normal dose of medication is used for the prevention, diagnosis, treatment of disease or regulation of physiological functions.

**FIGURE 1:**
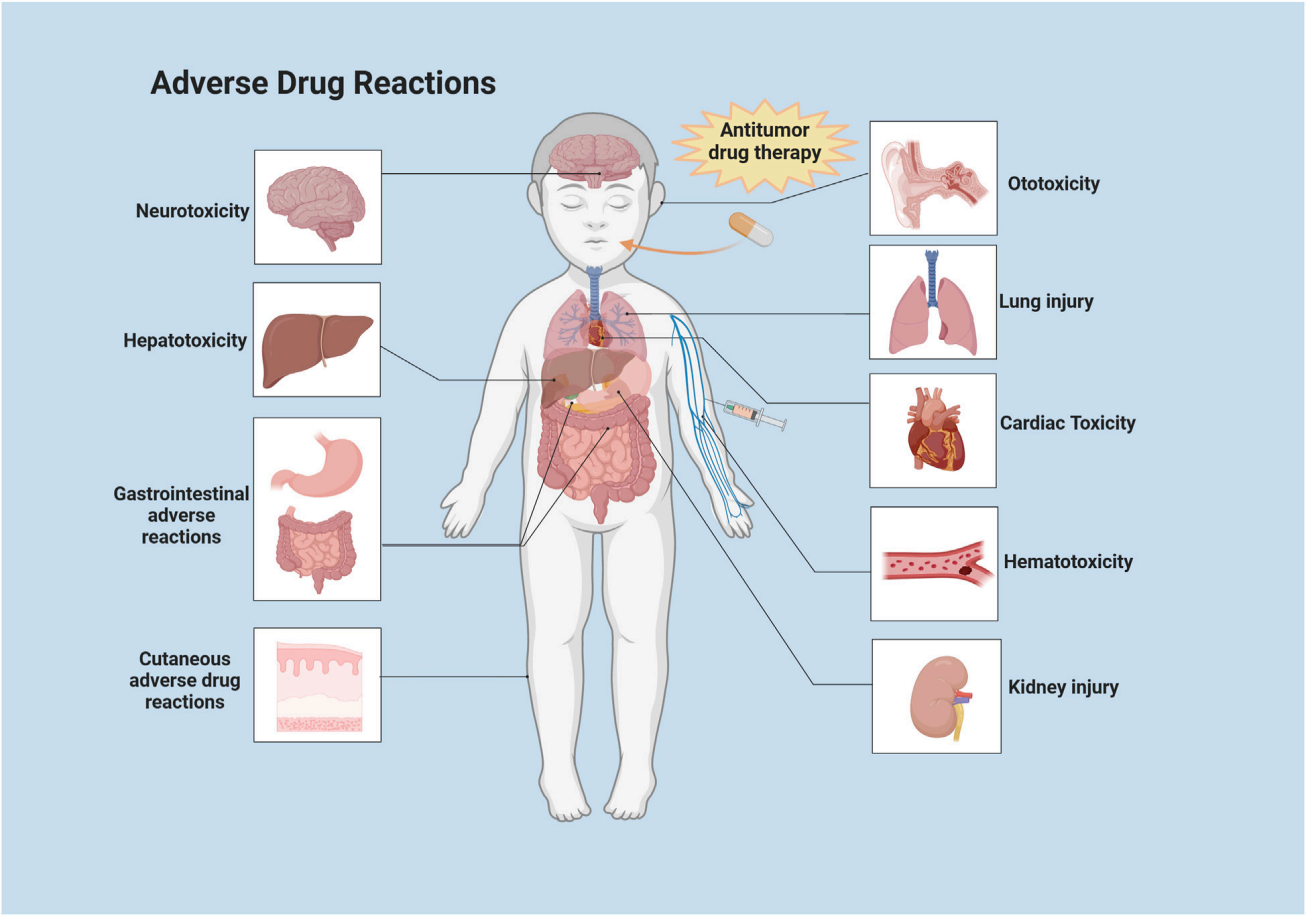
Systemic distribution of common ADRs to antitumor drugs in children. This illustration was created with BioRender (agreement number: XF2675PHGR).

Chemotherapy-related ADRs have become an important factor affecting cancer remission. Most ADRs are caused by chemotherapeutic agents, which damage the DNA of tumor cells and disrupt DNA replication in proliferating cells. However, many drugs fail to specifically target tumor tissues, resulting in ADRs causing damage to proliferating cells in healthy tissues. It was shown that the overall incidence of ADRs to medications among adults hospitalized for tumors was 8.37%, with 10.77% of severe ADRs (LAVAN et al., 2019). Among children hospitalized for tumors, the overall incidence of ADRs to medications was 9.53%, with severe ADRs accounting for 12.29% (IMPICCIATORE et al., 2001). In addition, over the course of drug therapy for children with tumors, 39.3% of ADRs that resulted in hospitalization were fatal (IMPICCIATORE et al., 2001). More than 21% of adult oncology patient visits were ADRs-related (LAVAN et al., 2019). Among pediatric cancer patients, 22% of hospitalizations were caused by ADRs and 44.2% of ADRs in general led to hospitalization (PAWAR et al., 2018). In recent years, there have been numerous domestic and international studies on ADRs in pediatric cancer patients, but few systematic summaries have been reported. In this article, we will review the ADRs associated with antitumor drugs in children and adolescent patients and the advances in strategies to cope with ADRs that consist of neurotoxicity, hematological toxicity, cardiotoxicity, ADRs of the respiratory system and gastrointestinal system and urinary system, ADRs of the skin and its adnexa, allergic reactions, and other ADRs (Figure 1). We also summarize countermeasures for managing ADRs in order to guide future research and clinical practices on the harmful adverse effects of anti-cancerous treatments in children.

## 2 Critical pediatric antitumor drugs

Anti-tumor drugs for children are classified according to their source and pharmacological mechanism, which include cytotoxic drugs, hormonal drugs, biological response modifiers, monoclonal antibodies, adjuvants, and other drugs. Based on their effects on the cell cycle, these drugs can also be categorized into cell cycle-specific and cell cycle-nonspecific drugs (Table 1 for details). The ADRs of antitumor drugs in children vary due to their wide variety and the different pharmacological effects of each drug. Different systems and organs in the body are affected in different ways, resulting in different clinical manifestations. Alkylating agents such as cyclophosphamide are more potent in killing M and G1-phase cells, but are less selective and also strongly toxic to normal cells (ABULFADL et al., 2023). Antimetabolites such as antifolates, antipurines, antipyrimidines, and cytarabine (Ara-C) are sensitive to S-phase cells and also function on G1-and G2-phase cells (AMARO-HOSEY et al., 2021; LI et al., 2022). Antitumor antibiotics such as zorubicin and doxorubicin (adriamycin) (ADM) form complexes with DNA and RNA and inhibit cell division (SKINNER et al., 1993). Anti-tumor phytoconstituents such as vincristine (VCR), vinblastine, and etoposide (VP-16) inhibit the formation of the spindle during cell division, causing mitosis to terminate at mid-cycle (GUTIéRREZ-GUTIéRREZ et al., 2010; SAŁAT, 2020a; MODY et al., 2020; LI et al., 2023). In addition to traditional chemotherapeutic drugs, some emerging therapeutic drugs such as targeted drugs (including anti-angiogenesis targeted drugs), immune checkpoint inhibitors (ICIs), monoclonal antibodies and adoptive cell therapy (ACT) (including chimeric antigen receptor (CAR) T cell (CAR-T) therapy and CAR natural killer cell (CAR-NK) therapy) have also expanded the range of cancer treatments (TURGAY et al., 2020).

**TABLE 1:** Classification of the main pediatric antitumor drugs.

Classification	Categories	Mechanism of action	Pharmaceuticals
Classification by source and pharmacological mechanism	Cytotoxic drugs	Effects on the chemical structure of DNA	Alkylating agents: Nitrogen mustard (HN2), Cyclophosphamide (CTX), Isocyclophosphamid (IFO), Thiotepa (TSPA), Nitrosoureas and methanesulfonates (Busulfan,BUS/BSF) (ABULFADL et al., 2023)
			Platinum compounds: Cisplatin (DDP), Carboplatin (CBP), Oxaliplatin (L-OHP) (JIN et al., 2021)
			Mitomycin (MMC) (BURZYNSKI, 2006)
			ADM, Epirubicine (EPI), Pirarubincin (THP-adriamycin) (SKINNER et al., 1993)
		Influence on nucleic acid synthesis	Dihydrofolate reductase inhibitor: Methotrexate (MTX), Pemetrexed (PEM) (LOPEZ-LOPEZ et al., 2020)
			Thymidine synthase inhibitor: Fluorouracil (5-Fu), Capecitabine (CAP) (HUANG et al., 2018)
			Purine nucleoside synthase inhibitor: Mercaptopurine (6-MP), Thioguanine (6-TG) (AMARO-HOSEY et al., 2021)
			Nucleotide reductase inhibitor: Hydroxyurea (HU) (PAWAR et al., 2018)
			DNA polymerase inhibitors: Ara-C, Gemcitabine (GEM) (AMARO-HOSEY et al., 2021)
		Effects on Nucleic Acid Transcription	Actinomycin D (ACD), Aclarubicin (aclacinomycin) (ACLA), Plicamycin (mithramycin) (MTH) (PAWAR et al., 2018)
		Topoisomerase inhibitors	Irinotecan (CPT-11), Topotecan (TPT), Hydroxycamptothecin (HCPT), VP-16, Teniposide (VM-26) (MODY et al., 2020)
	Hormones	Predominantly acts in mitotic M phase and interferes with microtubule protein synthesis	Paclitaxel (Taxol) (TAX, PTX), Vinblastine (VLB), Homoharringtonine (HH) (FLAHERTY et al., 2014)
		Other cytotoxic drugs	L-Asparaginase (L-ASP) (HONG et al., 2019)
		Antiestrogen	Tamoxifen (TAM), Toremifene (TOR), Exemestane (EXE) (PAWAR et al., 2018)
		Aromatase inhibitors	Aminoglutethimide (AG), Formestane (FMT), Letrozole (LTZ/LET), Anastrozole (ANA) (PAWAR et al., 2018)
		Progesterone	Medroxyprogesterone (MPA), Megestrol (Megace) (MA) (ZHANG et al., 2020)
		Sex hormone	Androgen: Methyltestosterone, Testosterone Propionate (TP)
			Oestrogen: Diethylstilbestrol (IMAN et al., 2017)
		Antiandrogen	Flutamide (TODD and LIPPARD, 2009)
		Luteinizing hormone-releasing hormone agonist/antagonist	Goserelin (Loanword), Leuprolide acetate (IMAN et al., 2017)
	Biological Response Modifier (BRC)		Interferon (IFN)、Interleukin-2(IL-2), Thymopeptides (FLAHERTY et al., 2014)
	Monoclonal Antibody		Rituximab (Mab Thera) (RTX), Cetuximab (C225), Trastuzumab Emtansine (T-DM1), Bevacizumab (BEV), PD-1, PD-L1 (TURGAY et al., 2020)
	Other	Cell differentiation/apoptosis inducer	Tretinoin (ATRA), Arsenous acids (PAWAR et al., 2018)
		Neoangiogenesis inhibitors	Rh-Endostatin (YH-16) (NG et al., 2014)
		Epidermal growth factor receptor inhibitor	Gefitinib, Erlotinib (FU et al., 2018)
	Adjuvant	Hematotropic drugs	Granulocyte Colony-Stimulating Factor (GCSF), Granulocyte-Macrophage Colony-Stimulating Factor (GMCSF), Interleukin-11(IL-11), Recombinant human erythropoietin (EPO) (MARTINO et al., 2018)
		Anti-nausea drug	Endansetron, Granisetron hydrochloride (PHILLIPS et al., 2016)
		Analgesic	Aspirin (APC), Paracetamol, Codeine, Tramadol, Morphine, Fentanyl Transdermal (IŻYCKI et al., 2016)
		Osteoclast inhibiting drugs	Bisphosphonate, Pamidronate (ZHU et al., 2020)
Classification by effect on the cell cycle	Cell cycle-specific drugs	M-phase	Vindesine (VDS), VCR, VLB, Vinorelbine (NVB/VRN), HCPT, Docetaxel (TXT, DTX), PTX (GUTIéRREZ-GUTIéRREZ et al., 2010; SAŁAT, 2020a)
		G1-phase	ASP, Adrenocorticotropic Hormone (ADH) (HONG et al., 2019)
		G2-phase	Bleomycin (BLM), Pingyangmycin (PYM) (YU et al., 2016)
		S-phase	Ara-C, GEM, 5-Fu, Ftorafur (FT-207), 6-MP、MTX, HU (AMARO-HOSEY et al., 2021)
	Cell cycle non-specific drugs	Alkylating agents	BUS, HN2, DDP, CTX (ABULFADL et al., 2023)
		Nitrosoureas	Carmustine (BCNU), Lomustine (CCNU), Streptozotocin (STZ) (PAWAR et al., 2018)
		Antimicrobials	Actinomycin D (ACTD), Daunorubicin (DNR), ADM, MMC (PAWAR et al., 2018)
		Other	Procarbazine (PCZ/PCB), Dacarbazine (DTIC) (PAWAR et al., 2018)

## 3 Antitumor therapy and ADRs in the nervous systems

Chemotherapy-induced neuropathy (CIN) by oncology therapeutic agents in children is one of the most common ADRs. CIN can affect the central nervous system or peripheral nervous system, with chemotherapy-induced peripheral neuropathy (CIPN) accounting for a predominant percentage of cases (SAŁAT, 2020b).

Common chemotherapeutic agents that induce neurotoxicity are platinum-based, such as L-OHP and DDP, PTX, and VLB (GUTIéRREZ-GUTIéRREZ et al., 2010; SAŁAT, 2020a). DDP and CBP may cause neurotoxicity by affecting dorsal root ganglion neurons and peripheral nerves through the accumulation of DNA complexes and inhibition of DNA repair processes. It is characterized by nerve ending impairment, optic nerve papillae edema and retrobulbar optic neuritis, and damage to the auditory nerve, which in severe cases can lead to irreversible high-frequency hearing loss (JIAO et al., 2021). Of the platinum-based chemotherapeutic agents, L-OHP has the most pronounced neurotoxicity, including acute and cumulative neurotoxicity. The acute manifestation is mainly characterized by abnormal and delayed sensation in the hands, feet and perioral area. The accumulation is mainly characterized by impaired walking due to delayed fine motor skills and/or impaired organoleptic sensation (SAŁAT, 2020a). Taxanes provoke neurotoxicity by acting on neuronal microtubules, causing destruction and demyelination of nerve axons (IŻYCKI et al., 2016). CIPN is characterized by peripheral bilateral sensory abnormalities, whereas chemotherapy-induced central neuropathy is characterized by developmental cognitive deficits and encephalopathy. VDS causes neurotoxicity mainly by affecting microtubules, which leads to defective axonal transport (FU et al., 2018). Neurotoxicity mainly causes autonomic and peripheral sensory-motor neuropathy, which manifests as numbness or a tingling sensation starting from the tips of the fingers (toes) that progresses centrally, accompanied by weakening or loss of deep tendon reflexes. In severe conditions, it can lead to muscle weakness, especially in the distal hand and foot (DUAN et al., 2018).

**FIGURE 2:**
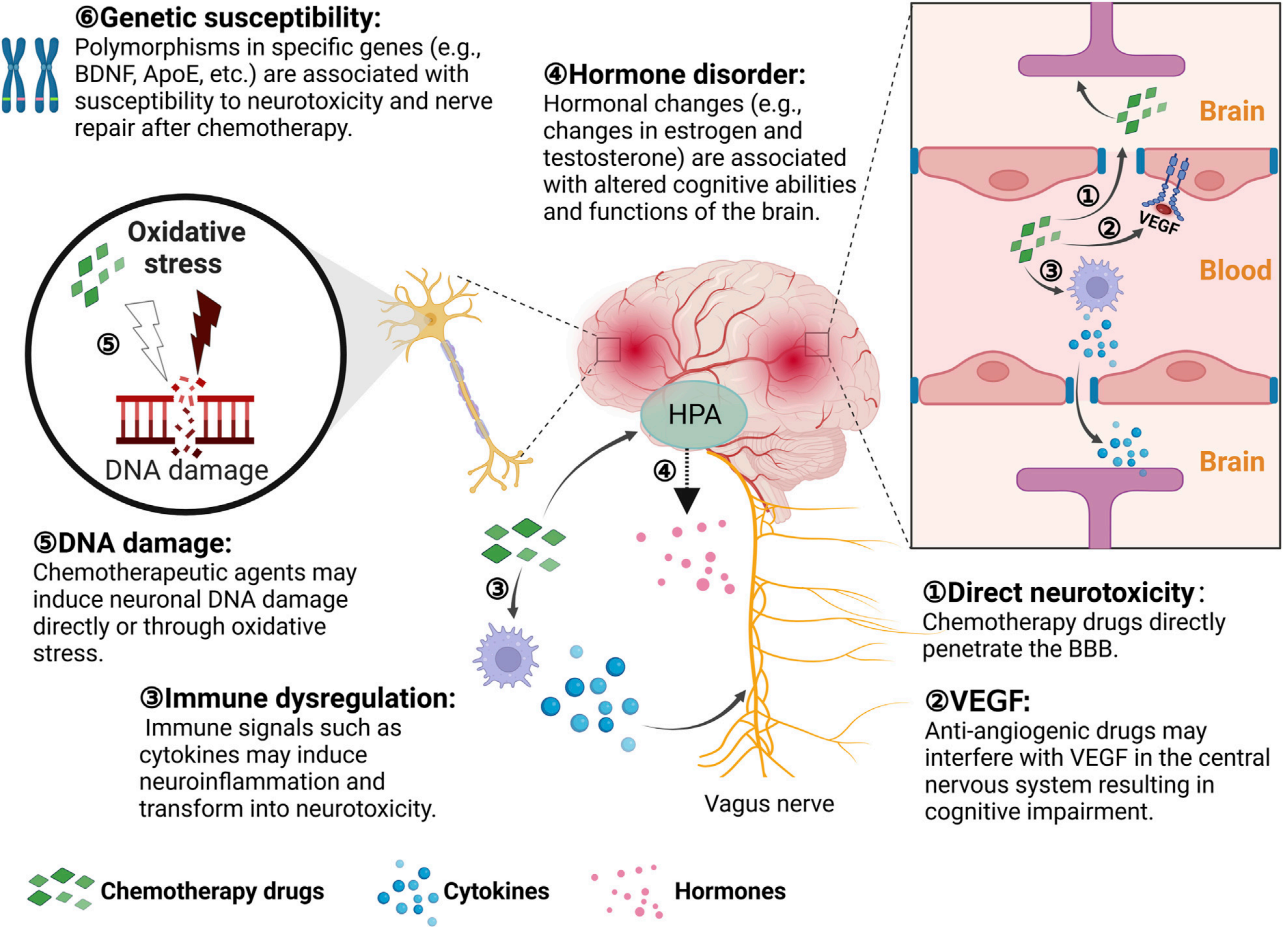
Potential neurotoxic pathways of chemotherapy in children. HPA: Hypothalamus-Pituitary-Adrenal; BBB: Blood-Brain-Barrier; VEGF: Vascular Endothelial Growth Factor. This illustration was created with BioRender (agreement number: YP26CY8TOS).

Targeted antitumor drugs mainly target tumor cells with little effect on normal cells, leading to fewer reports of neurotoxicity caused by targeted therapy for pediatric tumors. However, vascular endothelial growth factor (VEGF) prevents new blood vessel formation and also plays an important role in the central nervous system. Anti-angiogenic drugs achieve their antitumor effects by inhibiting vascular endothelial growth factor. Therefore, the use of anti-angiogenic drugs may interrupt vascular endothelial growth factors in the central nervous system, leading to cognitive impairment (NG et al., 2014). Cytokines and hormone level changes induced by anticancer drugs can also cause neurotoxicity by altering the hypothalamus-pituitary-adrenal (HPA). The potential pathways by which chemotherapy can cause neurotoxicity in children are shown in Figure 2. Targeted drugs that commonly cause neurotoxicity include thalidomide, pazopanib, C225, and bortezomib. ICIs have been widely applied in the treatment of various pediatric malignant tumors. ICIs have been found to affect different organs and tissues by disrupting the normal immune system and immune tolerance, leading to a variety of immune-related adverse events (irAEs) (MICHOT et al., 2020). These irAEs can also include neurologic immune-related adverse events, which are relatively infrequent (moderate to severe incidence of about 1%), but are extremely serious (MANCONE et al., 2018). Besides, ACT has been found to trigger neurotoxicity in children (ClinicalTrials.gov number: NCT01593696). The CD19/CD28ζ CAR-T cell-based therapies have caused neurotoxicity (e.g., confusion and aphasia) in some individuals during the treatment of children and adolescents with B-cell malignancies. This is closely related to cytokine release syndrome (CRS) and its severity (SHALABI et al., 2022). In pediatric and young adult patients with relapsed/refractory CD22 malignancies, CAR-T cell therapies targeting CD22 as an alternative to anti-CD19 resulted in CRS in 86.2% of patients, and transient mild neurotoxic symptoms in majority of patients (ClinicalTrials.gov number: NCT02315612) (SHAH et al., 2020).

## 4 Antitumor therapy and ADRs in the hematologic system

The hematologic system is the most common organ/system involved in ADRs in children treated with antineoplastic drugs. The main clinical manifestation is myelosuppression, which is a universal dose-limiting toxicity of antineoplastic agents. Leukopenia or/and neutropenia, anemia, and thrombocytopenia are the main clinical features of ADRs caused by antineoplastic agents (PAWAR et al., 2018). Among adverse effects, leukopenia, especially neutropenia, is the main cause of death from chemotherapy.

The vast majority of antineoplastic drugs possess a toxic effect on the hematopoietic system to varying degrees. The sensitivity of various blood cells in the bone marrow to chemotherapeutic agents depends on the length of their half-life. The half-life of white blood cells is only 6 h, the half-life of platelets is 5–7 days, and the half-life of red blood cells reaches up to 115 days (FRANCO, 2012). Drugs that damage DNA exhibit strong myelosuppressive effects, followed by those that inhibit RNA synthesis, whereas drugs that affect protein synthesis have the least effect. Representative drugs for myelosuppressive toxicity are Ara-C, MTX, CTX, anthracyclines, HN2, MMC, VM-26, VCR, TPT, TXT, DTX, PTX, GEM, DDP, CBP, IFO, VCR, CPT-11, and others. Ara-C is an antimetabolic drug that targets the proliferative phase of cells and is known to interrupt cell proliferation by arresting DNA synthesis. It is clinically utilized to treat acute leukemia, having the best efficacy for acute granulocytic leukemia, and its main ADR consists of myelosuppression (AMARO-HOSEY et al., 2021). MTX is an antifolate drug that hinders tumor growth by inhibiting dihydrofolate reductase. Clinically, it is mostly used for treating acute leukemia, head and neck tumors, bone tumors in children, and its main ADRs consists of gastrointestinal reactions and bone marrow suppression (ClinicalTrials.gov number: NCT00137111) (LOPEZ-LOPEZ et al., 2020). Bone marrow suppression and incidence of gastrointestinal disorders are more frequent due to the heavier use of Ara-C and MTX, as leukemia accounts for the highest proportion of childhood tumors. Therefore, hematopoietic function should be monitored during the application of Ara-C and MTX to avoid serious ADRs.

Aside from myelosuppression, ADRs of antineoplastic drugs on the hematologic system includes leukopenia due to infections (ZHU et al., 2020). In order to minimize post-chemotherapy infections, clinical attention to prevention is more important than treatment when granulocytopenia is present. The defense mechanisms that preserve the patient include reducing the invasion of new pathogens in the environment, reducing traumatic damage to defense mechanisms, and suppressing or killing pathogenic bacteria already present in the body. The series of hematologic ADRs introduced by ACT found in clinical studies should not be underestimated as well. CRS is the most common toxicity associated with CAR-T-related therapies, and even in rare cases it develops into the life-threatening fulminant HLH, which is characterized by anaemia, thrombocytopenia, leukopenia and neutropenia (NEELAPU et al., 2018). And CD22-targeting CAR-T cell therapy also resulted in HLH-like manifestations in 32.8% of children and young adults with relapsed/refractory malignancies (ClinicalTrials.gov number: NCT02315612) (SHAH et al., 2020).

## 5 Antitumor therapy and ADRs in the cardiac system

Many pediatric antineoplastic drugs produce toxic effects on the myocardium within the course of treatment, mainly manifested by changes in cardiac rhythm, altered blood pressure, ischemia, abnormalities in cardiac systolic or diastolic function, and delayed progressive cardiomyopathy in a minority of children. These ADRs are either temporary episodes or persistently present. Myocardial ADRs may be subclinical manifestations, but when severe they can be life-threatening and should be emphasized in clinical management. There are a number of chemotherapeutic drugs that cause cardiotoxicity including anthracyclines, PTX, CTX, 5-Fu, MTX, Ara-C, IFN, and monoclonal antibodies.

Cardiomyopathy is one of the earliest reported manifestations of cardiovascular toxicity caused by anthracycline chemotherapeutic agents, a drug commonly utilized in the treatment of leukemia (DE BAAT et al., 2022). Anthracycline-induced cardiovascular toxicity is of type I. One of the possible mechanisms of toxicity is attributed to the inhibition of topoisomerase II activity (TRIPAYDONIS et al., 2019). Topoisomerases are biological enzymes that are essential for biological activities and functions during DNA transcription, replication and recombination (TRIPAYDONIS et al., 2019). Anthracycline-induced cardiovascular toxicity is categorized according to the acuteness of its effects. Acute cardiovascular toxicity is rare, occurring in less than 1% of children at the time of administration or within 1 week after administration, and is characterized by a transient decline in myocardial contractility. Conversely, chronic cardiovascular toxicity occurs more commonly and is further categorized at early onset (within 1 year of dosing) versus late onset (more than 1 year after the end of dosing) (HU et al., 2018). Patients receiving anthracycline-based chemotherapy in childhood are prone to delayed cardiotoxicity when exposed to stressful situations, surgical procedures, or pregnancy during the formative years. The likely mechanism by which PTX causes cardiovascular toxicity is the mediation of massive histamine release. In animal experiments, researchers have found that stimulation of histamine receptors in cardiac tissue resulted in conduction disturbances and arrhythmias. In addition, PTX also affects cellular organelles, thereby causing myocardial injury (SCHIMMEL et al., 2004).

High doses of CTX have been reported to cause cardiovascular toxicity with clinical manifestations consisting of pericardial effusion, pericarditis, reversible decrease in cardiac contractility, and heart failure. Upon activation, CTX forms alkylated molecules that are able to bind to DNA, which triggers intra- and interstrand DNA breaks, leading to inhibition of DNA replication and enhanced apoptosis (ABULFADL et al., 2023). MTX occasionally causes syncope, myocardial infection, and arrhythmia and the most common ADRs related to Ara-C are pericarditis, pericardial effusion, and cardiac tamponade (STRICKLAND and VEY, 2022). GEM is an alternative option applied in recent years for the treatment of refractory or recurrent pediatric solid tumors. Cardiotoxicity, including ventricular arrhythmias and reduced left ventricular ejection fraction, occasionally leading to atrial fibrillation, has been observed in some patients (BENDER et al., 2023). ADRs of IFNs and ILs on the cardiovascular system include ischemia and infarction, arrhythmias, and cardiomyopathy (SONNENBLICK and ROSIN, 1991). Long-term IFN use carries a high risk of reduced left ventricular ejection fraction and congestive heart failure (SONNENBLICK and ROSIN, 1991). High doses of IL-2 are associated with unfavorable cardiovascular and hemodynamic effects, similar to infectious shock and cardiotoxic reactions (JOHN-PAUL and BARTELS, 2017). As a new generation of therapy that has emerged in recent years, monoclonal antibodies are commonly used in the treatment of lymphoma and certain hematological tumors in children. However, these drugs tend to cause massive cytokine release, leading to hypertension, fever, dyspnea, hypoxia and even death (HONG and DIOUN, 2014).

## 6 Antitumor therapy and ADRs in the respiratory

Although the incidence of respiratory adverse events when using antitumor drugs in clinical practice is relatively low, the consequences are often serious and even fatal. Anticancer drugs that frequently cause pulmonary toxicity include BLM, MTX, Ara-C, CTX, BCNU, and VCR. Pulmonary adverse reactions are clinically divided into two categories: allergic and pulmonary fibrosis (BALDO et al., 2015). The former mainly consists of allergic pneumonitis, mostly comprising of acute attacks. The clinical manifestations of pulmonary fibrosis are generally late, usually appearing weeks to months or even years after halting drug use. The main symptoms include dyspnea, chest tightness, dry cough, fatigue and discomfort. At the onset of drug administration, the lung tissue undergoes a series of complex changes progressively at the subcellular molecular level and exhibits different clinical signs ranging from asymptomatic to lethal pulmonary fibrosis. The molecular basis of this is related to pathological changes in the cellular matrix, which includes collagen, plasma fiber-binding proteins and elastin in the lung tissue, in response to antitumor drugs. The large deposition of extracellular matrix components is caused by the expansion of fibroblasts and the transfer of cells capable of matrix production from other sites to the lungs due to the chemotactic effect of peptides, as well as the elevated level of matrix synthesis in lung cell populations (BENNETT et al., 2007). Antineoplastic drug-induced pulmonary fibrosis was strongly associated with the number of doses, dosages, combinations, and even the timing and rate of drug use.

Pulmonary injury is the most serious and common adverse reaction of BLM, ranging from non-specific pneumonia to fatal pulmonary fibrosis, with a mortality rate as high as 50% (YU et al., 2016). There is evidence that combination with other cytotoxic drugs further increases the pulmonary toxicity of BLM (GALM et al., 2011). The potential mechanisms of pulmonary toxicity caused by BLM include: ⅰ) Production of reactive oxygen metabolites that directly damage lung tissue. ⅱ) Massive infiltration of leukocytes and increased release of proteases. ⅲ) Proliferation of fibroblasts, which increases collagen synthesis (YU et al., 2016). In addition to causing hypersensitivity pneumonitis, MTX also has a strong direct toxic effect on lung tissue and can easily cause lung damage, but rarely pulmonary fibrosis (GALM et al., 2011). The pulmonary damage of Ara-C manifests as pulmonary edema and is related to the frequency of administration. When high doses were clinically applied to treat acute leukemia, 22% of children developed subacute pulmonary failure, with the first signs of toxicity occurring at an average of 6 d (2–21 d) after initiation of treatment (YANG et al., 2023). CTX and IFO may injure lung tissue through the production of reactive oxygen metabolites. The incidence of CTX-induced lung injury is less than 1%, in a non-dose-dependent manner, with subacute episodes ranging from 3 weeks to 8 years after drug administration (CAMPAGNE et al., 2020). BCNU can cause acute and even delayed pulmonary fibrosis (BURZYNSKI, 2006). Vincristine analogs (VLB, VDS, VCR) have been shown to cause adverse pulmonary effects, but the mechanism of injury is unclear. Most reports state that their combination with MMC or CTX and ADM frequently triggers subacute pulmonary toxic effects with symptoms of respiratory distress (ClinicalTrials.gov number: NCT00006237) (FLAHERTY et al., 2014).

## 7 Antitumor therapy and ADRs in the digestive system

Digestive system ADRs are the most common adverse reactions during chemotherapy for malignant tumors, mainly manifesting as loss of appetite, nausea, vomiting, acute gastritis, mucositis, abdominal pain, diarrhea, and constipation. These ADRs not only affect the quality of life of the child directly, but also tend to impede the smooth implementation of chemotherapy and drug dosage, and even become life-threatening in severe cases. Common highly emetogenic agents include DDP, CTX, and BCNU. The emetogenic risk classification of different chemotherapeutic agents is shown in Table 2.

**TABLE 2:** Emetogenic risk classification of different chemotherapeutic agents^a^.

High >90%	Moderate 30%–90%	Low 10%–30%	Minimal <10%
Anthracycline/CTX >1,500 mg/m^2^	Anthracycline/CTX ≤1,500 mg/m^2^	PTX	C225
	IFO/DDP <50 mg/m^2^	TXT, DTX (IV&Oral)	T-DM1
	CBP/L-OHP >75 mg/m^2^	Albumin-bound PTX	BEV
DDP ≥50 mg/m^2^	CPT-11	GEM, 5-Fu	Gefitinib
		ADM liposomal	Sorafenib
		PEM, VP-16	CAP

^a^ According to the National Comprehensive Cancer Network (NCCN) Antiemetic Guidelines: For nausea and vomiting caused by a combination of multiple medications, the regimen should be based on the medication with the highest risk of causing vomiting.

Nausea and vomiting are the most frequent toxic side effects of chemotherapy drugs on the digestive system (KARPMAN and KURZROCK, 2004). Chemotherapeutic drugs cause nausea and vomiting through two main pathways: Injuries to the GI mucosa leads to the release of substances from enterochromaffin cells, such as 5-hydroxytryptamine (5-HT), which stimulates 5-HT_3_ receptors on afferent vagus nerves in the intestinal wall and excites the vomiting center in the medulla oblongata. Alternatively, chemicals cause vomiting by acting on the emetic chemoreceptor zone (CTZ) in the fourth ventricle of the brain (JIN et al., 2021). DDP is one of the most emetogenic chemotherapeutic agents to date, and the types of nausea and vomiting caused by the drug are usually categorized into five types based on the time of onset: acute, delayed, anticipatory, eruptive, and refractory (JIN et al., 2021). Chemotherapeutic agents cause acute or persistent diarrhea in approximately 10% of children, with diarrhea rates ranging from 50% to 80%, especially with 5-Fu and CPT-11 containing regimens (CASANOVA et al., 2016). Among them, CPT-11 causes delayed diarrhea and L-OHP causes neurogenic diarrhea. Diarrhea occurs mainly as a result of an imbalance in the secretion and absorption functions of the small intestine caused by acute damage to the intestinal mucosa from chemotherapeutic drugs. Severe diarrhea predisposes to fatal dehydration, acute renal insufficiency and electrolyte disturbances. Mucositis with neutropenia can be further aggravated by secondary intestinal and systemic infections (MODY et al., 2020).

Oral mucositis and ulcers are another common GI reaction to chemotherapy (HONG et al., 2019). Chemotherapeutic agents that predispose to mucositis are predominantly antimetabolites including MTX, Ara-C, 5-Fu, CAP, and VP-16. Mucositis is one of the dose-limiting toxicities of 5-Fu and MTX. The mechanism by which chemotherapeutic agents evoke oral mucositis is twofold: direct damage to the mucosa and secondary localized infection (MAUNEY et al., 2022). Bone marrow transplantation and high-dose chemotherapy with graft-versus-host disease (GVHD) and some cytokines such as IL-1 and TNF-α are also involved in the mucosal damage process. The reduction of neutrophils after chemotherapy tends to exacerbate secondary localized infections of ulcers with anaerobic, aerobic bacteria and even mycobacteria (HONG et al., 2019). Oral mucositis tends to appear within 2–4 days after chemotherapy, which intensifies over the next week and then gradually moves into a healing phase. Chemotherapeutic agents that tend to cause abdominal pain are usually found in vincristine analogs, deoxyfluorouracil, VM-26, DNR, Ara-C, RTX, T-DM1, etc., Medications that are prone to constipation mainly include VLB, PTX, VP-16, DDP, etc., In addition, loss of appetite is also a common adverse reaction of most chemotherapy drugs. Hepatotoxicity and liver injury are also common and unwanted adverse reactions occurring in patients due to chemotherapeutic agents such as MTX, BUS/BSF, PTX, CTX, PTX, 5-Fu, and MMC (KARPMAN and KURZROCK, 2004). Toxic reactions in the liver are mainly hepatocellular dysfunction, veno-occlusive liver disease and chronic liver fibrosis. ASP, which is routinely applied in childhood leukemia, is susceptible to hepatic abnormalities and acute pancreatitis (MAUNEY et al., 2022).

## 8 Antitumor therapy and ADRs in the urinary system

Damage to the urinary system from antineoplastic agents occurs mainly as a result of renal parenchymal injury and urinary tract irritation reactions (CRONA et al., 2017). Renal damage includes abnormal renal function, elevated serum creatinine or proteinuria, oliguria or anuria, and acute renal failure. Medications that lead to renal parenchymal injury include MTX, DDP, MMC, and BUS/BSF, among which DDP in particular is the most nephrotoxic and dose-limiting. DDP has been found to be predisposed to renal tubular epithelial necrosis, and in severe cases, acute renal failure (JIN et al., 2021). MTX readily forms crystals in normal acidic urine, which contributes to urinary tract obstruction and tubular damage. The clinical features of cytotoxicity are cystitis, including urinary frequency, urgency, dysuria and hematuria, and bladder fibrosis (SKINNER et al., 1993). IFO, CTX, and HCPT have been shown to lead to urinary tract irritation and hemorrhagic cystitis, which manifests as difficulty in urination, frequent and painful urination, and in severe cases produces hematuria. These symptoms appear over a period of hours or weeks and eventually subside within a few days of withdrawal.

## 9 Antitumor therapy and ADRs in the skin and its appendages

Dermal toxicity mainly refers to local skin damage, rash, alopecia, and other direct or indirect damage to the skin and its appendages caused by antitumor drugs (STRUMIA et al., 2022). Antitumor drugs will damage the skin tissue locally to different degrees upon extravasation from the blood vessels to the surrounding tissues (STRUMIA et al., 2022). In mild cases, it can trigger localized pain and phlebitis, whereas in severe cases, it can lead to localized skin blisters, ulcers, tissue detachment, and even lead to dysfunction. For example, anthracyclines, MMC, and VCR induces severe skin necrosis; BLM, platinum, 5-FU, TXL, and MTZ induce moderate damage; and L-ASP, BCNU, DTIC, MEL, TSPA, and MTX can cause mild skin irritation. The management of dermal reactions to toxicity caused by drug extravasation lies in careful infusion, close observation, and prompt detection and treatment of leakages. Most antitumor drugs can cause skin rashes to varying degrees (NG et al., 2018). The most common ADR, of targeted drugs such as gefitinib and C225, is skin rash. These rashes are mainly located on the face and upper trunk, are non-pruritic, and may resolve spontaneously.

The ADR that CAP predisposes to is hand-foot syndrome, which is manifested by warmth, pain, and redness of the fingers (toes), and in severe cases, may progress to desquamation, ulceration, and severe pain (WRIGHT et al., 2015). Although alopecia is also a common ADR of antineoplastic drugs, most of them are reversible. Hair regeneration usually occurs within 1 month–2 months after drug withdrawal, and no additional treatment is required. If necessary, a scalp tourniquet or ice cap cooling can be used to reduce the amount of medication that reaches hair follicles, thereby reducing hair loss (NG et al., 2018). In addition, certain drugs can cause nail deformities, such as CTX, BLM, TXT, DTX, ADM, 5-Fu, and paxillin (STRUMIA et al., 2022).

## 10 Antitumor therapy and allergic reactions and other ADRs

The incidence of allergic reactions to antineoplastic drugs in children is 5%–15% (GIAVINA-BIANCHI et al., 2017). Clinical manifestations include rash, angioneurotic edema, dyspnea, hypotension, anaphylactic shock, etc., (TURGAY et al., 2020). Common drugs that cause allergic reactions include L-ASP, PYM, BLM, PTX, anthracyclines, platinum drugs, etc., among which allergic reactions caused by L-ASP and TXL are more frequent. For chemotherapeutic drugs that have a high incidence and severity of allergic reactions, antiallergic pretreatment must be performed regardless of the dose and duration of titration. In the case of allergic manifestations, administration of the drug should be stopped immediately, and a combination of H_1_ and H_2_ receptor antagonists should be used for symptomatic relief. Supplementation with glucocorticoids, antihypertensive agents or bronchodilators may also be appropriate (GIAVINA-BIANCHI et al., 2017). RTX, T-DM1, and BEV are also susceptible to immediate hypersensitivity (BALDO and PHAM, 2013). In addition, pain and skin redness may occur at the venous sites where antitumor agents are injected, and long-term use of the drugs will lead to skin pigmentation, striated hardening of the veins and venous embolism (LYLE et al., 2015). Chemotherapeutic drugs that tend to cause venous damage includes HN, 5-FU, NVB, anthracyclines, etc.

In addition to the common ADRs of antineoplastic drugs in children as outlined above, hearing loss, dysgenitalism, gynecomastia and early menarche or amenorrhea in girls may occur. Long-term toxic effects including reproductive dysfunction, carcinogenesis and teratogenesis may also occur. Studies have shown that 22%–74% of pediatric oncology patients developed permanent hearing damage after DDP treatment (FREYER et al., 2020). DDP ototoxicity severely reduces the quality of life of children, especially affecting early language development and social cognitive ability. Numerous studies have confirmed that DDP leads to caspase-3-activated apoptosis in auditory hair cells by inducing elevated reactive oxygen species (RAMKUMAR et al., 2021). Mammalian auditory hair cells, once damaged, are permanently inactive with no capacity for spontaneous regeneration. A large number of animal experiments have confirmed that platinum drugs can cause a series of irreversible health damage, such as reproductive system damage, DNA damage, and chromosomal aberrations (TODD and LIPPARD, 2009; ZHANG et al., 2020; IBRAHIM et al., 2021). Toxic effects of DDP on the uterus and ovaries and irreversible genotoxicity have been demonstrated in animals (ZHANG et al., 2020; IBRAHIM et al., 2021).

## 11 Similarities and differences in ADRs to antineoplastic drugs in adults and children

Antitumor drugs may cause ADRs in children such as growth inhibition and infertility. The ADRs for antineoplastic drugs in adults and children involve more similar systems but differ in their specific manifestations. For example, when treated with RTX, rare but serious events—described primarily in adults—include progressive multifocal encephalopathy, prolonged neutropenia, and fatal viral reactivation (MCATEE et al., 2021). Children were susceptible to infusion reactions with an incidence of 50%–90% and adults had an incidence of 4%–15% (SALLES et al., 2017). The incidence of infection in children was 40%–50%, which was higher than that in adults (26%–35%) (FLEURY et al., 2016). Children were also more likely to be immunosuppressed compared to adults (about 50% incidence in children compared to 20%–30% in adults) (HIRANO et al., 2014). ASP was an effective drug for acute lymphoblastic leukemia (ALL) in children, and in recent years it had been increasingly used for adults over 22 years of age. The main risk factors for hepatotoxicity due to ASP involved older age and weight gain, and thus adults were more prone to hepatotoxicity than children during treatment (ALDOSS et al., 2022). The incidence of pancreatitis was higher in adults (about 10%) than in children (about 5%) (Stock et al., 2019). However, children were more prone to allergic reactions (GUPTA et al., 2020b). CRS occurred in both adults and children during treatment with immunotherapy, but occurred more frequently in children (about 77%) than in adults (about 50%) (RAGOONANAN et al., 2021). Children were also more prone to neurologic adverse effects, with a higher incidence of electroencephalogram (EEG) changed (about 83.3%) than adults (about 76%) (GUST et al., 2019). Given the differences in response to antitumor drugs between children and adults, child-specific standards and criteria should be established in clinical practice to enhance safety monitoring and effectiveness evaluation.

## 12 Measures to address ADRs to antitumor drugs

To mitigate the unfavorable impact of ADRs on the treatment and quality of life of children, a range of preventive and responsive measures have been implemented clinically. To prevent local tissue necrosis caused by extravasation of chemotherapeutic drugs, the vascular condition of the child should be assessed before intravenous injection, and intravenous access should be established with saline. Drugs should be diluted to a certain concentration, the drip rate should be controlled when titrating, and the stimulating drugs need to be preferentially infused in the case of multidrug combination therapy (GOOLSBY and LOMBARDO, 2006). Children should be instructed to protect the blood vessels, and localized pressure should be applied for 5–7 min after needle removal. In cold weather, the limb on the side of the puncture is immersed in warm water before performing venipuncture to dilate the blood vessels and facilitate puncture (LV et al., 2021). In case of extravasation, infusion must be stopped immediately, followed by limitation of limb movement, retraction of extravasated drug, needle removal, and the application of antagonistic drugs or local closure measures in severe cases (GOOLSBY and LOMBARDO, 2006). Cold or hot compresses should also be applied when appropriate.

Little benefit is currently available for the treatment of drug-mediated neurotoxicity. In symptomatic individuals, the duration of intravenous infusion may be extended. Potentially effective drugs include gabapentin, duloxetine, and VB_6_ tablets (IŻYCKI et al., 2016). Peripheral neurotoxicity is reversible, and in severe cases, chemotherapy needs to be stopped first. Intermittent supplementation of large amounts of B vitamins is beneficial to alleviate peripheral neuritis (FU et al., 2018). To tackle hematologic toxicity led by antitumor drugs, prevention should be emphasized to reduce the risk of infection. Protective isolation measures can be taken to maintain the sterility of the ward room utilizing air purification equipment. When broad-spectrum antibiotics are administered, the medication needs to be adjusted based on the drug sensitivity results. Granulocyte colony-stimulating factor can also be administered to alleviate hematologic toxicity, and discontinuation is indicated by leukocytes over 10 × 10^9^/L (MARTINO et al., 2018). Thrombopoietin and IL-11 need to be administered in cases of low platelet counts, and monoclonalized platelets should be transfused in cases of severe platelet drop (ZHU et al., 2020).

For the prevention and treatment of cardiotoxicity, the main concern is to be vigilant against anthracyclines. Dexpropylenimine is the only agent that is available to prevent anthracycline cardiotoxicity (DE BAAT et al., 2022). For co-administration, anthracyclines are given first followed by PTX to minimize cardiotoxicity from their interaction (SCHIMMEL et al., 2004). Concomitant treatment with anthracyclines and T-DM1 should be avoided (HU et al., 2018). For the management of respiratory ADRs, the total dose of the drug needs to be controlled, with the total dose of BLM not exceeding 400 mg (YU et al., 2016). Immediate discontinuation of medication and symptomatic management is the first action in response to pulmonary toxicity. Oxygen intake is not allowed at high levels when treating with hormones and antibiotics. Antiemetic drugs are prescribed when nausea and vomiting occur with chemotherapy. Currently, commonly available antiemetic drugs include: dopamine receptor blockers, such as metoclopramide and domperidone; phenothiazines, such as isoprinosine, chlorpromazine, and phenazopyridine; adrenocorticotropic hormones, such as dexamethasone; antihistamines, such as phenylephrine; 5-HT_3_ receptor antagonists, such as paroxetine, granisetine, and tolstanesetron; NK-1 receptor antagonists, such as aripipipramine; and benzodiazepines, such as diazepam (PHILLIPS et al., 2016). It is also necessary to prevent water and electrolyte imbalance. Octreotide represents one of the preferable drugs available for alleviating diarrhea caused by oncological chemotherapy (WU et al., 2008). It improves electrolyte absorption by reducing intestinal motility and digestive fluid secretion, thus reducing stool volume. Receiving folic acid and vitamin B_12_ supplementation in conjunction with PEM therapy is effective in preventing or reducing treatment-related hematologic or gastrointestinal reactions (ClinicalTrials.gov identifier: NCT00520936) (WARWICK et al., 2013). In order to prevent or minimize nephrotoxicity caused by antitumor drugs, patients should drink adequate amounts of water to accelerate drug elimination, and have a light diet to avoid the formation of uric acid aggravated by heavy meats. Hydration and alkalinization of the urine are required when administering highly nephrotoxic drugs such as DDP and MTX (JIN et al., 2021). When taking high doses of MTX, calcium folinate is available to alleviate ADRs (SKINNER et al., 1993). Systemic administration of sodium thiosulfate is expected to reduce toxicity and improve efficacy when high-dose DDP is given intracavitally for the treatment of malignant pleural and ascites (CRONA et al., 2017). Moreover, amphotericin has been shown to prevent or minimize the nephrotoxicity of DDP (CRONA et al., 2017). Mescaline sodium should be supplemented concurrently to prevent cystitis that may result from high-dose IFO and CTX therapy (SKINNER et al., 1993). To mitigate hand-foot syndrome due to CAP treatment, the duration of the drip can be shortened or switched to oral administration (HUANG et al., 2018). Furthermore, dermatitis can be controlled with anti-allergic drugs or hormones (LUPO et al., 2014). Targeted delivery of antitumor drugs via nanomaterials has also been reported to alleviate ADRs (CHOU et al., 2020; JINDAL et al., 2020; KURZĄTKOWSKA et al., 2021), but additional studies are needed for confirmation.

Efforts to address ADRs associated with ACT also continue to evolve. The production of CAR-T cells is time-consuming and hence fails to meet the therapeutic needs of rapidly progressing cancers. To shorten infusion time, the FasT CAR-T next-day manufacturing platform was developed (YANG et al., 2022). The novel anti-CD19 CAR-T cells based on this production platform are more proliferative and provide better anti-tumor efficacy and less cellular depletion in refractory/relapsed B-cell acute lymphoblastic leukemia (B-ALL) compared to conventional CAR-T therapy. However, in addition to multi-system ADRs, CAR-T cell therapies still face obstacles such as long production periods and the scarcity of patients' own T-cells available for mass production applications. NK cells, as a class of natural tumor-killing lymphocytes, have attracted much attention owing to their advantages over T cells, such as unrestricted allogeneic graft-versus-host disease (GVHD), short production periods, and non-antigen-dependent natural tumor-killing effects (XIE et al., 2020; LIU et al., 2021). Clinical trials (ClinicalTrials.gov number: NCT03056339) of CAR-NK therapies for hematologic malignancies have been conducted and no major toxicities including CRS, neurotoxicity and GVHD have been reported to date, suggesting that CAR-NK might be an alternative therapy to CAR-T (ZHANG et al., 2022). Although CAR-T and CAR-NK therapies, as a new generation of therapeutic methods to regulate immune cells as an entry point to fight against tumors, have achieved a good outcome in hematological tumors, their application in solid tumors is still greatly hindered by the lack of cancer-specific antigens, the inefficiency of immune cells transported to the tumor site, and the immunosuppressive microenvironment (PAN et al., 2022). More recently, CAR macrophages are being explored in an attempt to overcome the shortcomings of other cell therapies in the treatment of solid tumors as a new approach towards cell therapy (KLICHINSKY et al., 2020; PAN et al., 2022; CHEN et al., 2023).

**FIGURE 3:**
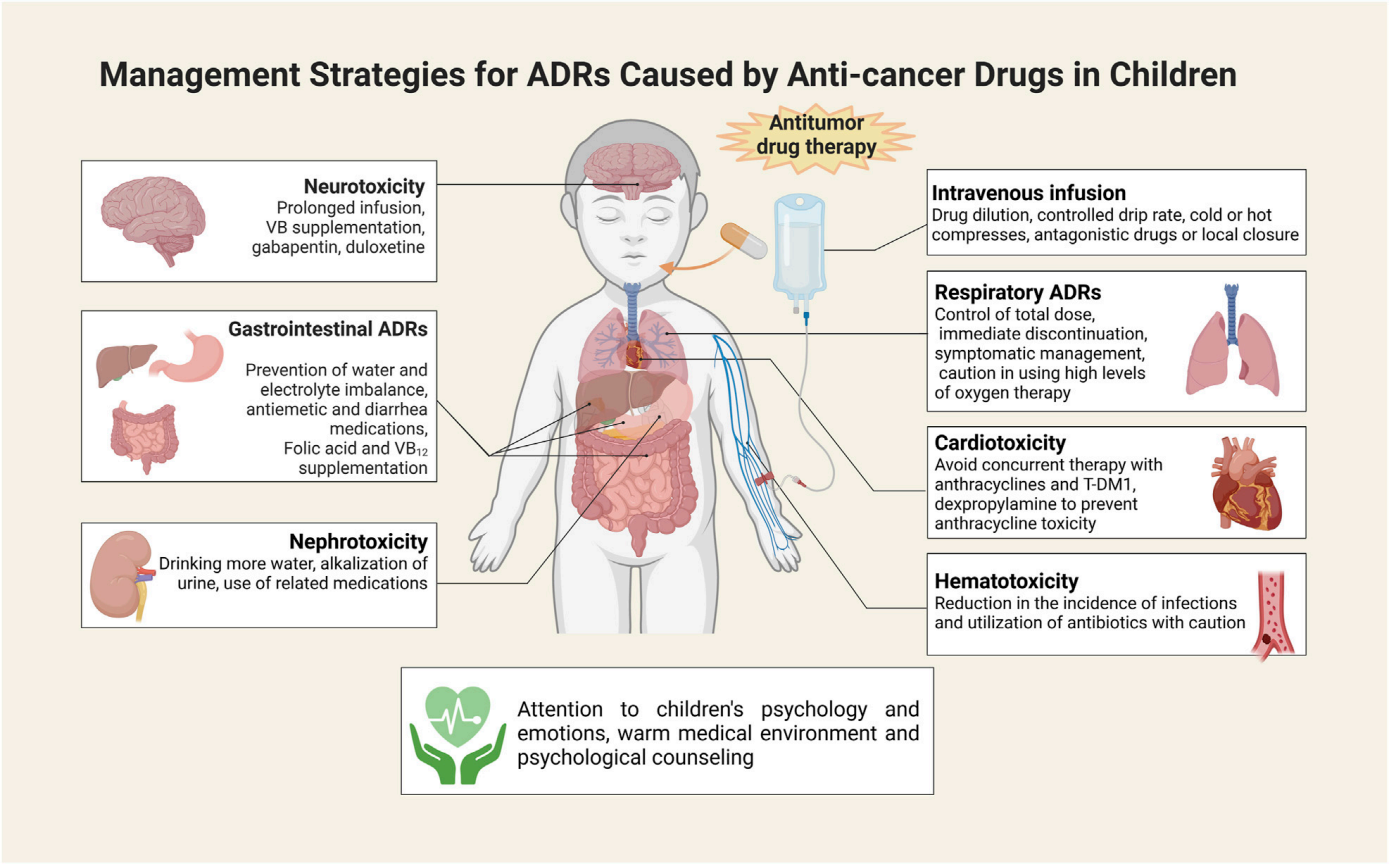
Measures to address ADRs to antitumor drugs in children. This illustration was created with BioRender (agreement number: IK26CU3ATC).

In addition to the above countermeasures, it is also critical for healthcare professionals and parents to pay close attention to the psychology and emotions of the children during treatment. Favorable psychological care measures are helpful for children to adapt to the new medical environment by influencing their state of mind and perceptions. On the other hand, psychological counseling helps create an optimal psychological state that is beneficial to the treatment and recovery process, and can help mitigate ADRs associated with chemotherapeutic drugs (Figure 3).

## 13 Conclusion and outlook

In summary, ADRs caused by antineoplastic drugs in children may involve multiple organs or systems. The top 3 common ADRs occur in the gastrointestinal system, the hematologic system, and the skin. As leukemia accounts for the highest proportion of pediatric tumors, Ara-C and MTX are highly utilized. Therefore, among all antineoplastic drugs, ADRs from these two drugs are the most frequent, followed by CTX and platinum compounds. The type and severity of ADRs to antineoplastic drugs varies depending on the drug and the individual, and the treatment varies as well. The general principle of management is prevention and symptomatic treatment. Reasonable dosages and treatment regimens according to the specific conditions of the children should be developed to avoid overuse and abuse when using these drugs. Meanwhile, the emergence of ADRs must be monitored closely and treatment programs should be promptly adjusted. Early prevention, close monitoring, and timely management are critical approaches to improving drug efficacy, reducing ADRs, and alleviating pain in children. Based on previous studies, we systematically summarized common ADRs of antineoplastic drugs in children and the measures to prevent or minimize ADRs. This review thus provides an informative basis for future studies and clinical applications.

Nevertheless, there are certain scientific problems and challenges that need to be addressed with regard to ADRs that are common in pediatric and adolescent oncology patients. Firstly, conducting clinical trials of drugs for children is very challenging due to their special physiological characteristics and ethical issues. How to design reasonable clinical trial protocols to ensure the accuracy and reliability of trial results is an urgent issue to be solved. Secondly, the construction of vigilance and risk management system for ADRs in children is not yet perfect, how to improve the level of pharmacovigilance, detection and treatment of ADRs in a timely manner is an important challenge at present. Finally, the individualized drug therapy for pediatric oncology patients is in high demand, and how to individualized drug therapy to reduce the risk of adverse effects according to children’s genes, age, weight and other factors is an important focus of future research.
